# High prevalence of p16 staining in malignant tumors

**DOI:** 10.1371/journal.pone.0262877

**Published:** 2022-07-21

**Authors:** Noémi De Wispelaere, Sebastian Dwertmann Rico, Marcus Bauer, Andreas M. Luebke, Martina Kluth, Franziska Büscheck, Claudia Hube-Magg, Doris Höflmayer, Natalia Gorbokon, Sören Weidemann, Katharina Möller, Christoph Fraune, Christian Bernreuther, Ronald Simon, Christian Kähler, Anne Menz, Andrea Hinsch, Frank Jacobsen, Patrick Lebok, Till Clauditz, Guido Sauter, Ria Uhlig, Waldemar Wilczak, Stefan Steurer, Eike Burandt, Rainer Krech, David Dum, Till Krech, Andreas Marx, Sarah Minner

**Affiliations:** 1 Institute of Pathology, University Medical Center Hamburg-Eppendorf, Hamburg, Germany; 2 Institute of Pathology, Clinical Center Osnabrueck, Osnabrueck, Germany; 3 Department of Pathology, Academic Hospital Fuerth, Fuerth Germany; University of Nebraska Medical Center, UNITED STATES

## Abstract

p16 (CDKN2A) is a member of the INK4 class of cell cycle inhibitors, which is often dysregulated in cancer. However, the prevalence of p16 expression in different cancer types is controversial. 15,783 samples from 124 different tumor types and 76 different normal tissue types were analyzed by immunohistochemistry in a tissue microarray format. p16 was detectable in 5,292 (45.0%) of 11,759 interpretable tumors. Except from adenohypophysis in islets of Langerhans, p16 staining was largely absent in normal tissues. In cancer, highest positivity rates were observed in uterine cervix squamous cell carcinomas (94.4%), non-invasive papillary urothelial carcinoma, pTaG2 (100%), Merkel cell carcinoma (97.7%), and small cell carcinomas of various sites of origin (54.5%-100%). All 124 tumor categories showed at least occasional p16 immunostaining. Comparison with clinico-pathological data in 128 vulvar, 149 endometrial, 295 serous ovarian, 396 pancreatic, 1365 colorectal, 284 gastric, and 1245 urinary bladder cancers, 910 breast carcinomas, 620 clear cell renal cell carcinomas, and 414 testicular germ cell tumors revealed only few statistically significant associations. Comparison of human papilloma virus (HPV) status and p16 in 497 squamous cell carcinomas of different organs revealed HPV in 80.4% of p16 positive and in 20.6% of p16 negative cancers (p<0.0001). It is concluded, that a positive and especially strong p16 immunostaining is a feature for malignancy which may be diagnostically useful in lipomatous, urothelial and possibly other tumors. The imperfect association between p16 immunostaining and HPV infection with high variability between different sites of origin challenges the use of p16 immunohistochemistry as a surrogate for HPV positivity, except in tumors of cervix uteri and the penis.

## Introduction

The p16 protein is encoded by the *cyclin dependent kinase inhibitor 2A* gene (*CDKN2A*, syn. *MTS-1*, *INK4a* or *p16*^*INK4*^) located at chromosome 9p21 [1]. p16 inhibits cell cycle progression from G1 to S phase [2] through binding and inactivating cyclin dependent kinases CDK4 and CDK6 [3]. In its cell cycle inhibiting function, p16 interplays with the retinoblastoma (RB1) and the p53 tumor suppressor genes. In case of an inactivation of p53 or RB1 and especially in case of inactivation of both proteins, p16 can be markedly upregulated. Accordingly, a particularly strong up-regulation is seen in human papilloma virus (HPV) infected cells, where both p53 and RB1 are inactivated by the HPV proteins E6 and E7 [4,5].

More than 3,000 studies have employed immunohistochemistry to study the role of p16 expression in normal and neoplastic tissues. Due to its strong overexpression in HPV infected cells, p16 immunohistochemistry is routinely used in diagnostic pathology as a surrogate parameter for HPV infection and a marker for HPV related anogenital and oropharyngeal neoplasia. The role of p16 expression in other cancer types is less clear. The rate of reported p16 positivity is highly variable for many tumors. For example, the fraction of p16 positive cases ranged from 43% to 100% in squamous cell carcinoma of the cervix [6,7], 9% to 98% in colorectal adenocarcinoma [8–10], 12% to 64% in hepatocellular carcinoma [11,12], 4% to 96% in malignant melanoma [13–15], 20% to 62% in mesothelioma [16–18], and 0% to 100% in liopsarcomas [19–21]. Although many studies have described a prognostic role of reduced or increased p16 expression, these results have often not been confirmed by others. In several cancer types including breast, prostate, ovarian, and colorectal cancer, both reduced expression [22–25] and overexpression [26–29] have been reported to be linked to poor prognosis. Altogether, these conflicting data are likely to be caused by the use of different antibodies, immunostaining protocols, and criteria to determine p16 positivity in these studies.

To better understand the role of p16 immunohistochemistry in different tumor types, a comprehensive study analyzing a large number of neoplastic and non-neoplastic tissues under highly standardized conditions is needed. We thus analyzed p16 expression in more than 15,000 tumor tissue samples from 124 different tumor types and subtypes as well as 76 non-neoplastic tissue types by immunohistochemistry in a tissue microarray (TMA) format.

## Materials and methods

### Tissue Microarrays (TMAs)

To study p16 expression in normal and neoplastic human tissues, preexisting TMAs containing 15,783 primary tumors from 124 tumor types and subtypes as well as 608 samples of 76 different normal tissues were used. Detailed histopathological data on grade, pT and pN status were available for 7,598 cancers (invasive breast carcinoma of no special type, colorectal carcinoma, endometroid endometrial carcinoma, clear cell renal cell carcinoma, serous high grade ovarian carcinoma, adenocarcinoma of the pancreas, adenocarcinoma of the stomach, germ cell tumors, carcinoma of the vulva and urinary bladder carcinoma). Clinical follow up data were available for 254 patients who had undergone cystectomy for muscle invasive (pT≥2) urinary bladder cancer (median follow-up time = 14 (range 1–77) months) and 978 patients with invasive breast carcinoma of no special type (median follow-up time = 50 (range 1–88) months). The composition of both normal and cancer TMAs is described in detail in the results section. All samples were derived from the archives of the Institute of Pathology, University Hospital of Hamburg, Germany, the Institute of Pathology, Clinical Center Osnabrueck, Germany, and the Department of Pathology, Academic Hospital Fuerth, Germany. Tissues were fixed in 4% buffered formalin and then embedded in paraffin. TMA tissue spot diameter was 0.6 mm. Informed patient consent was not required for this retrospective study. The use of anonymized archived remnants of diagnostic tissues for manufacturing of TMAs and their analysis for research purposes as well as patient data analysis has been approved by local laws (HmbKHG, §12) and by the local ethics committee (Ethics commission Hamburg, WF-049/09). All work has been carried out in compliance with the Helsinki Declaration.

### Immunohistochemistry

Freshly cut TMA sections were immunostained on one day and in one experiment. Slides were deparaffinized with xylol, rehydrated through a graded alcohol series and exposed to heat-induced antigen retrieval for 5 minutes in an autoclave at 121°C in pH 9 DakoTarget Retrieval Solution™ (Agilent, CA, USA; #S2367). Endogenous peroxidase activity was blocked with Dako Peroxidase Blocking Solution™ (Agilent, CA, USA; #52023) for 10 minutes. Primary antibody specific against p16 protein (rabbit recombinant clone MSVA-016R; MS Validated Antibodies GmbH, Hamburg, Germany) was applied at 37°C for 60 minutes at a dilution of 1:150. Bound antibody was then visualized using the EnVision Kit™ (Agilent, CA, USA; #K5007) according to the manufacturer’s directions. The sections were counterstained with haemalaun. For tumor tissues, the percentage of positive neoplastic cells was estimated, and the staining intensity was semiquantitatively recorded (0, 1+, 2+, 3+). For statistical analyses, the staining results were categorized into four groups. Tumors without any staining were considered as negative. Tumors with 1+ staining intensity in ≤70% of cells or 2+ intensity in ≤30% of cells were considered weakly positive. Tumors with 1+ staining intensity in >70% of cells, 2+ intensity in 31–70%, or 3+ intensity in ≤30% were considered moderately positive. Tumors with 2+ intensity in >70% or 3+ intensity in >30% of cells were considered strongly positive.

### HPV polymerase chain reaction (PCR) and sequencing

HPV status was analyzed in a subset of 551 squamous cell carcinomas including 80 oral, 60 pharyngeal, 60 laryngeal, 80 cervical, 30 vaginal, 80 vulvar, 80 penile, 40 skin and 41 anal canal tumors. Detection of HPV-DNA was performed on formalin-fixed, paraffin-embedded tumor specimens. One 4μm microtome section was taken from each sample for DNA extraction using the Maxwell® RSC DNA FFPE Kit (Promega, Fitchburg, WI, USA) according to the manufacturer’s protocol. Suitability of the isolated DNA for PCR analysis was verified by amplification of a ß-globin sequence with primers generating an amplicon of 217 bp (forward 5′-GCCATCACTAAAGGCACCGAGC-3′ and reverse 5′-TGGGCATGTGGAGACAGAGAAGA-3′). Detection of HPV was performed using primers HPV-GP 6+ (5´-GAAAAATAAACTGTAAATCATATTC-3´) and HPV-GP 5+ (5´-TTTGTTACTGTGGTAGATACTAC-3´) which generate amplicons ranging between 139–145 bp. The thermocycler protocol included initial denaturation at 95°C for 10 min, followed by 34 cycles of 95°C for 90 sec, 55°C for 90 sec and 72°C for 120 sec, and a final extension step at 72°C for 7 min. PCR products were visualized by standard agarose gel electrophoresis. Samples with negative ß-globin PCR were excluded from further analysis. Samples with positive ß-globin PCR but negative HPV PCR were reported as HPV-negative. Samples with positive ß-globin PCR and positive HPV PCR were reported as HPV-positive and subjected to bidirectional Sanger sequencing employing the Genetic Analyzer 3130 xl device (Applied Biosystems, Foster City, CA, USA) using primers GP 5+ and GP 6+. Sequences were analyzed with NCBI’s Basic Local Alignment Search Tool (BLAST) [30] to determine the HPV type.

### Statistics

Statistical calculations were performed with JMP14 software (SAS Institute Inc., NC, USA). Contingency tables and the chi^2^-test were performed to search for associations between p16 and tumor phenotype. Survival curves were calculated according to Kaplan-Meier. The Log-Rank test was applied to detect significant differences between groups. A p-value of ≤0.05 was considered as statistically significant.

## Results

### Technical issues

A total of 11,759 (74.5%) of 15,783 tumor samples and 405 (66.6%) of 608 normal samples were interpretable for p16 immunostaining in our TMA analysis. Non-interpretable samples (4,024; 25.5%) either lacked unequivocal tumor cells or were absent on the TMA.

### p16 in normal tissue

**Fig 1:**
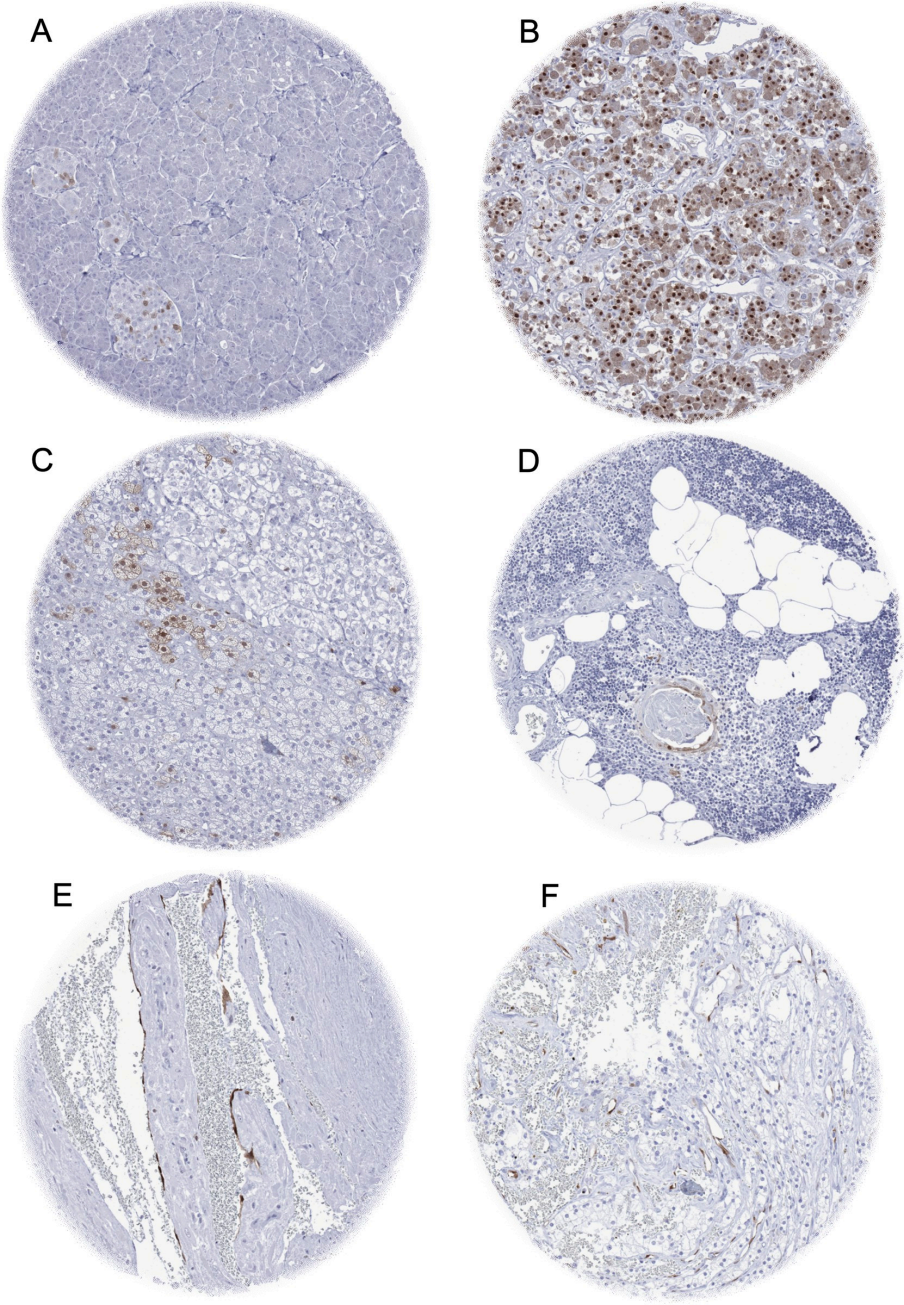
p16 immunostaining in non-neoplastic tissue. The panels show a nuclear and cytoplasmic p16 staining of a fraction of cells of pancreatic islets of Langerhans (A), a large fraction of epithelial cells in the adenohypophysis (B), a fraction of cells of corpuscles of Hassall‘s of thymus (C), and of scattered adrenocortical cells (D). A p16 positivity is also seen in endothelial cells of blood vessels in a normal placenta (E) and in an otherwise p16 negative clear cell carcinoma of the kidney (F).

p16 staining was strongest in islets of Langerhans of the pancreas (Fig 1A) and in a large fraction of cells in the adenohypophysis (Fig 1B). Positive staining was also found in a fraction of cells of corpuscles of Hassall‘s of thymus (Fig 1C), scattered adrenocortical cells (Fig 1D), and endothelial cells of blood vessels in a normal placenta (Fig 1E) and in an otherwise p16 negative clear cell carcinoma of the kidney (Fig 1F).

p16 immunostaining was absent in endothelium and media of the aorta, the heart, striated muscle, tongue muscle, myometrium of the uterus, muscular wall of the appendix, esophagus, stomach, ileum, colon descendens, kidney pelvis, and urinary bladder, corpus spongiosum of the penis, ovarian stroma, fat, skin, hair follicle and sebaceous glands of the skin, oral mucosa of the lip, oral cavity, surface epithelium of the tonsil, transitional mucosa and skin of the anal canal, ectocervix, squamous epithelium of the esophagus, urothelium of the kidney pelvis and urinary bladder, amnion and chorion of the mature placenta, spleen, antrum and corpus of the stomach, epithelium of the gallbladder, liver, Brunner gland of the duodenum, cortex and medulla of the kidney, seminal vesicle, epididymis, testis, lung, endocervix, mucosa of the fallopian tube, decidua of the early placenta, in the cerebellum, and white and grey matter of the cerebrum.

### p16 immunostaining in tumor cells

**Fig 2:**
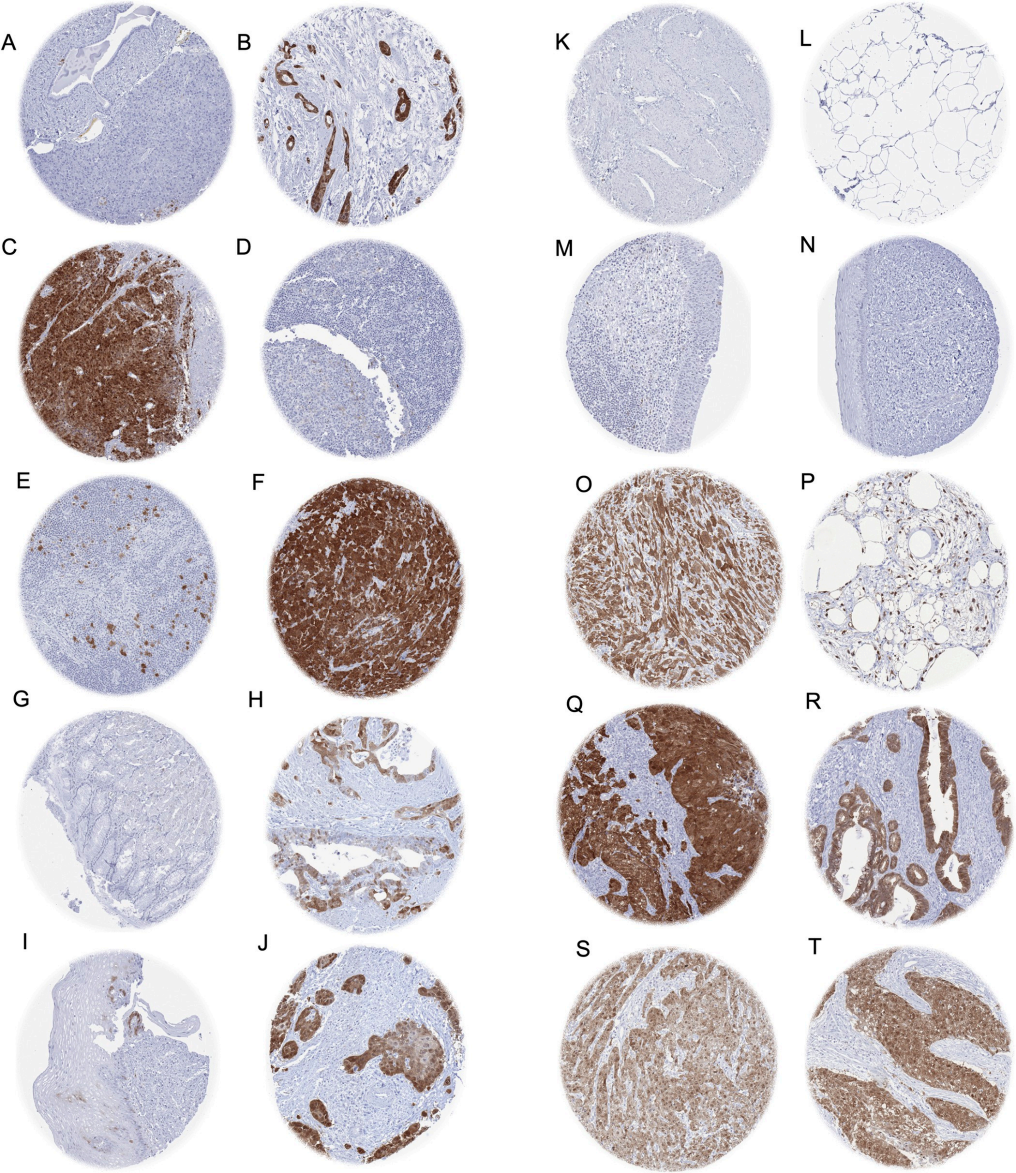
p16 immunostaining of tumors and related normal tissues. In the pancreas, a moderate p16 immunostaining is regularly seen in a subset of islet cells and only occasionally occurs in few scattered cells of excretory ducts (A), but p16 expression can be strong in cases of ductal adenocarcinoma (B) and of neuroendocrine carcinoma (C). In normal lymphatic tissues, a weak p16 staining occurs in germinal centre macrophages and in some scattered lymphocytes (D) but a strong staining is seen in neoplastic cells of some Hodgkin‘s (E) and diffuse large B-cell lymphomas (F). In the stomach, few normal epithelial cells may show p16 staining (G) while p16 staining can be strong in gastric adenocarcinoma (H). In the esophagus, few cells with weak to moderate p16 staining can be found in some samples of normal squamous epithelium (I) but p16 staining can be intense in squamous cell carcinoma (J). p16 immunostaining is usually absent in normal myometrium (K), fat (L), urothelium (M), and cervical squamous epitheium (N) while staining can be intense in tumors derived from these tissues such as leimyosarcoma of the uterus (O), liposarcoma (P), urothelial carcinoma (Q) as well as adenocarcinoma (R) and squamous cell carcinoma (S) of the uterine cervix. A similarly strong p16 staining can also be seen in other squamous cell carcinomas such as of the skin (T).

**Fig 3:**
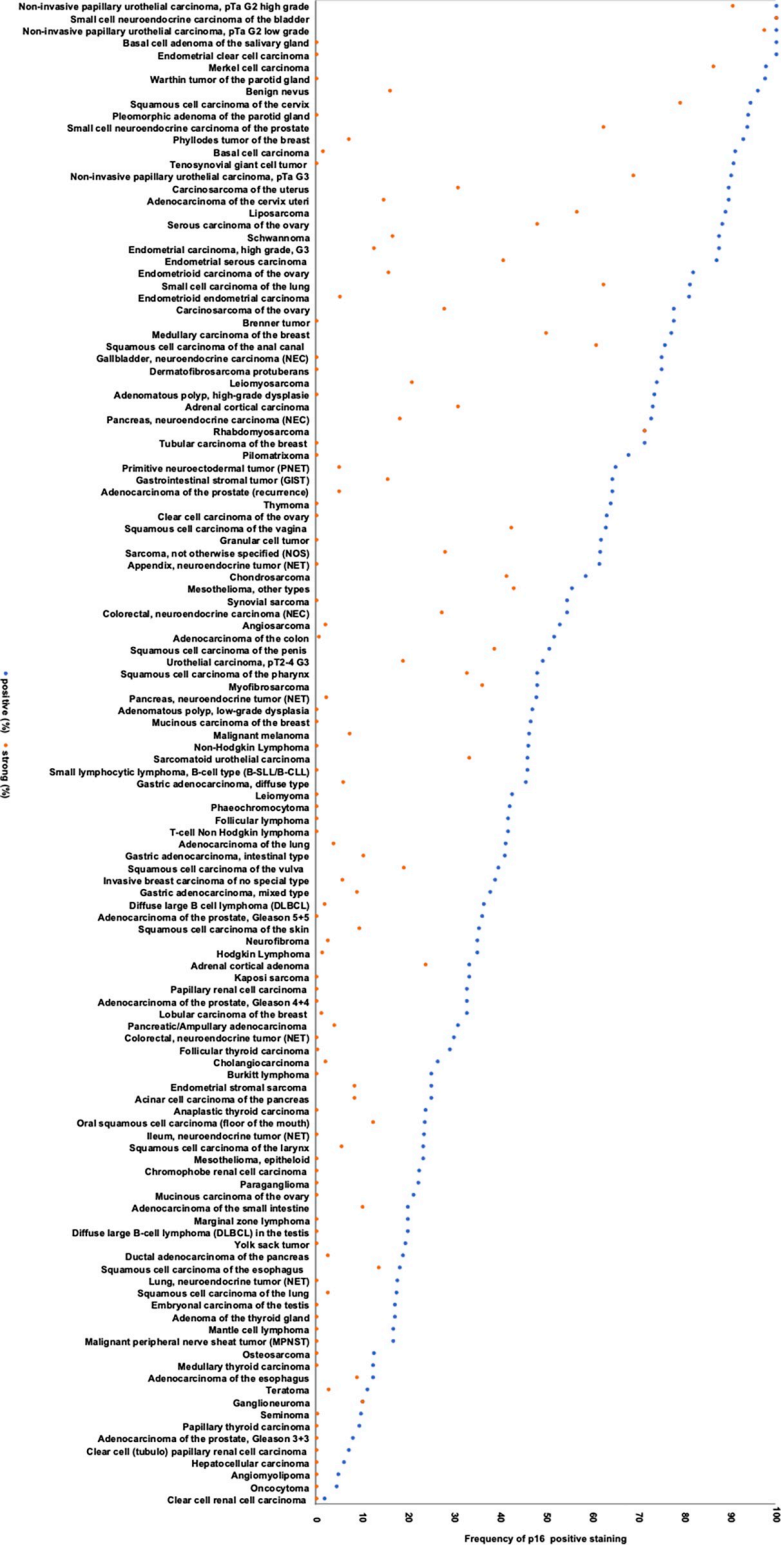
Ranking order of p16 immunostaining in human tumors. Both the frequency of positive cases (blue dots) and the frequency of strongly positive cases (orange dots) is shown.

Positive p16 immunostaining was detectable in 5,292 (45.0%) of the 11,759 analyzable tumors, including 3,152 (26.8%) with weak, 683 (5.8%) with moderate, and 1,457 (12.4%) with strong immunostaining. The staining pattern was heterogenous and included cases with variable percentage of positive tumor cells as well as tumors with predominantly cytoplasmic and predominantly nuclear staining. Representative images of p16 positive tumors are shown in Fig 2. All 124 tumor categories showed a detectable p16 expression in at least one case with 71 (57.3%) tumor categories showing at least one case with strong positivity (Table 1). A comparison between p16 expression in normal tissues und the corresponding tumor types is given in S2 Table. A graphical representation of a ranking order of p16 positive and strongly positive cancers is given in Fig 3.

**Table 1:** p16 immunostaining in human tumors.

			p16 immunostaining					
	tumor entity	on TMA (n)	analyzable (n)	negative (%)	weak (%)	moderate (%)	strong (%)	positive (%)
**Tumors of the skin**	Pilomatrixoma	35	28	32.1	64.3	3.6	0.0	67.9
	Basal cell carcinoma	88	67	9.0	68.7	20.9	1.5	91.0
	Benign nevus	29	25	4.0	60.0	20.0	16.0	96.0
	Squamous cell carcinoma of the skin	90	85	64.7	20.0	5.9	9.4	35.3
	Malignant melanoma	48	41	53.7	19.5	19.5	7.3	46.3
	Merkel cell carcinoma	46	44	2.3	2.3	9.1	86.4	97.7
**Tumors of the head and neck**	Squamous cell carcinoma of the larynx	110	90	76.7	13.3	4.4	5.6	23.3
	Squamous cell carcinoma of the pharynx	60	52	51.9	7.7	7.7	32.7	48.1
	Oral squamous cell carcinoma (floor of the mouth)	130	114	76.3	7.9	3.5	12.3	23.7
	Pleomorphic adenoma of the parotid gland	50	33	6.1	81.8	12.1	0.0	93.9
	Warthin tumor of the parotid gland	49	41	2.4	87.8	9.8	0.0	97.6
	Basal cell adenoma of the salivary gland	15	13	0.0	100.0	0.0	0.0	100.0
**Tumors of the lung, pleura and thymus**	Squamous cell carcinoma of the lung	77	40	82.5	5.0	10.0	2.5	17.5
	Adenocarcinoma of the lung	200	107	58.9	27.1	10.3	3.7	41.1
	Small cell carcinoma of the lung	20	16	18.8	12.5	6.3	62.5	81.3
	Mesothelioma, epitheloid	39	30	76.7	23.3	0.0	0.0	23.3
	Mesothelioma, other types	76	63	63.5	12.7	0.0	42.9	55.6
	Thymoma	29	25	36.0	60.0	4.0	0.0	64.0
**Tumors of the female genital tract**	Squamous cell carcinoma of the vagina	78	73	37.0	11.0	9.6	42.5	63.0
	Squamous cell carcinoma of the vulva	130	116	60.3	12.1	8.6	19.0	39.7
	Squamous cell carcinoma of the cervix	130	125	5.6	4.0	11.2	79.2	94.4
	Adenocarcinoma of the cervix uteri	50	48	10.4	52.1	22.9	14.6	89.6
	Endometrioid endometrial carcinoma	236	195	19.0	63.6	12.3	5.1	81.0
	Endometrial serous carcinoma	82	69	13.0	26.1	20.3	40.6	87.0
	Carcinosarcoma of the uterus	48	39	10.3	20.5	38.5	30.8	89.7
	Endometrial carcinoma, high grade, G3	13	8	12.5	62.5	12.5	12.5	87.5
	Endometrial clear cell carcinoma	8	5	0.0	80.0	20.0	0.0	100.0
	Endometrial stromal sarcoma	12	12	75.0	16.7	0.0	8.3	25.0
	Endometrioid carcinoma of the ovary	115	89	18.0	47.2	19.1	15.7	82.0
	Serous carcinoma of the ovary	567	446	11.7	24.0	16.4	48.0	88.3
	Mucinous carcinoma of the ovary	97	66	78.8	18.2	3.0	0.0	21.2
	Clear cell carcinoma of the ovary	54	38	36.8	47.4	15.8	0.0	63.2
	Carcinosarcoma of the ovary	47	36	22.2	30.6	19.4	27.8	77.8
	Brenner tumor	9	9	22.2	66.7	11.1	0.0	77.8
**Tumors of the breast**	Invasive breast carcinoma of no special type	1387	960	61.1	29.5	3.6	5.7	38.9
	Lobular carcinoma of the breast	294	168	67.3	30.4	1.2	1.2	32.7
	Medullary carcinoma of the breast	26	22	22.7	13.6	13.6	50.0	77.3
	Tubular carcinoma of the breast	27	14	28.6	71.4	0.0	0.0	71.4
	Mucinous carcinoma of the breast	58	30	53.3	40.0	6.7	0.0	46.7
	Phyllodes tumor of the breast	50	42	7.1	71.4	14.3	7.1	92.9
**Tumors of the digestive system**	Adenomatous polyp, low-grade dysplasia	50	49	53.1	44.9	2.0	0.0	46.9
	Adenomatous polyp, high-grade dysplasie	50	49	26.5	63.3	10.2	0.0	73.5
	Adenocarcinoma of the colon	1882	1434	48.2	48.6	2.6	0.6	51.8
	Adenocarcinoma of the small intestine	10	10	80.0	0.0	10.0	10.0	20.0
	Gastric adenocarcinoma, diffuse type	176	101	54.5	28.7	10.9	5.9	45.5
	Gastric adenocarcinoma, intestinal type	174	127	59.1	23.6	7.1	10.2	40.9
	Gastric adenocarcinoma, mixed type	62	45	62.2	28.9	0.0	8.9	37.8
	Adenocarcinoma of the esophagus	133	57	87.7	1.8	1.8	8.8	12.3
	Squamous cell carcinoma of the esophagus	124	44	81.8	0.0	4.5	13.6	18.2
	Squamous cell carcinoma of the anal canal	91	87	24.1	4.6	10.3	60.9	75.9
	Cholangiocarcinoma	130	102	73.5	20.6	3.9	2.0	26.5
	Hepatocellular carcinoma	50	50	94.0	6.0	0.0	0.0	6.0
	Ductal adenocarcinoma of the pancreas	612	410	81.2	13.2	3.2	2.4	18.8
	Pancreatic/Ampullary adenocarcinoma	89	52	69.2	21.2	5.8	3.8	30.8
	Acinar cell carcinoma of the pancreas	13	12	75.0	8.3	8.3	8.3	25.0
	Gastrointestinal stromal tumor (GIST)	50	45	35.6	40.0	8.9	15.6	64.4
**Tumors of the urinary system**	Non-invasive papillary urothelial carcinoma, pTa G2 low grade	177	116	0.0	0.0	2.6	97.4	100.0
	Non-invasive papillary urothelial carcinoma, pTa G2 high grade	141	106	0.0	0.9	8.5	90.6	100.0
	Non-invasive papillary urothelial carcinoma, pTa G3	187	132	9.8	6.1	15.2	68.9	90.2
	Urothelial carcinoma, pT2-4 G3	1214	732	50.7	20.4	10.1	18.9	49.3
	Small cell neuroendocrine carcinoma of the bladder	18	18	0.0	0.0	0.0	100.0	100.0
	Sarcomatoid urothelial carcinoma	25	24	54.2	12.5	0.0	33.3	45.8
	Clear cell renal cell carcinoma	858	648	98.2	1.7	0.2	0.0	1.8
	Papillary renal cell carcinoma	255	129	67.2	31.3	1.6	0.0	32.8
	Clear cell (tubulo) papillary renal cell carcinoma	21	13	92.9	7.1	0.0	0.0	7.1
	Chromophobe renal cell carcinoma	131	80	77.7	22.3	0.0	0.0	22.3
	Oncocytoma	177	128	95.5	4.5	0.0	0.0	4.5
**Tumors of the male genital organs**	Adenocarcinoma of the prostate, Gleason 3+3	83	63	92.1	6.3	1.6	0.0	7.9
	Adenocarcinoma of the prostate, Gleason 4+4	80	64	67.2	31.3	1.6	0.0	32.8
	Adenocarcinoma of the prostate, Gleason 5+5	85	61	63.9	36.1	0.0	0.0	36.1
	Adenocarcinoma of the prostate (recurrence)	330	284	35.6	57.0	2.5	4.9	64.4
	Small cell neuroendocrine carcinoma of the prostate	17	16	6.3	18.8	12.5	62.5	93.8
	Seminoma	620	454	90.3	9.3	0.2	0.2	9.7
	Embryonal carcinoma of the testis	50	41	82.9	14.6	2.4	0.0	17.1
	Yolk sack tumor	50	36	80.6	19.4	0.0	0.0	19.4
	Teratoma	50	36	88.9	5.6	2.8	2.8	11.1
	Squamous cell carcinoma of the penis	80	75	49.3	8.0	4.0	38.7	50.7
**Tumors of endocrine organs**	Adenoma of the thyroid gland	50	47	83.0	17.0	0.0	0.0	17.0
	Papillary thyroid carcinoma	114	96	90.6	9.4	0.0	0.0	9.4
	Follicular thyroid carcinoma	392	333	70.9	26.4	2.4	0.3	29.1
	Medullary thyroid carcinoma	158	130	87.7	11.5	0.8	0.0	12.3
	Anaplastic thyroid carcinoma	107	80	76.3	22.5	1.3	0.0	23.8
	Adrenal cortical adenoma	45	42	66.7	2.4	7.1	23.8	33.3
	Adrenal cortical carcinoma	26	26	26.9	23.1	19.2	30.8	73.1
	Phaeochromocytoma	50	50	58.0	38.0	4.0	0.0	42.0
	Appendix, neuroendocrine tumor (NET)	22	13	38.5	53.8	7.7	0.0	61.5
	Colorectal, neuroendocrine tumor (NET)	10	10	70.0	30.0	0.0	0.0	30.0
	Ileum, neuroendocrine tumor (NET)	49	47	76.6	23.4	0.0	0.0	23.4
	Lung, neuroendocrine tumor (NET)	19	17	82.4	17.6	0.0	0.0	17.6
	Pancreas, neuroendocrine tumor (NET)	102	96	52.1	40.6	5.2	2.1	47.9
	Colorectal, neuroendocrine carcinoma (NEC)	11	11	45.5	0.0	27.3	27.3	54.5
	Gallbladder, neuroendocrine carcinoma (NEC)	4	4	25.0	0.0	75.0	0.0	75.0
	Pancreas, neuroendocrine carcinoma (NEC)	13	11	27.3	36.4	18.2	18.2	72.7
**Tumors of haemotopoetic**	Hodgkin Lymphoma	103	77	64.9	23.4	10.4	1.3	35.1
**and lymphoid tissues**	Non-Hodgkin Lymphoma	62	52	53.8	44.2	1.9	0.0	46.2
	Small lymphocytic lymphoma, B-cell type (B-SLL/B-CLL)	50	48	54.2	45.8	0.0	0.0	45.8
	Diffuse large B cell lymphoma (DLBCL)	114	110	63.6	29.1	5.5	1.8	36.4
	Follicular lymphoma	88	84	58.3	41.7	0.0	0.0	41.7
	T-cell Non Hodgkin lymphoma	24	24	58.3	33.3	8.3	0.0	41.7
	Mantle cell lymphoma	18	18	83.3	16.7	0.0	0.0	16.7
	Marginal zone lymphoma	16	15	80.0	20.0	0.0	0.0	20.0
	Diffuse large B-cell lymphoma (DLBCL) in the testis	16	15	80.0	13.3	6.7	0.0	20.0
	Burkitt lymphoma	5	4	75.0	25.0	0.0	0.0	25.0
**Tumors of soft tissue and bone**	Tenosynovial giant cell tumor	45	43	9.3	88.4	2.3	0.0	90.7
	Angiomyolipoma	91	84	95.2	4.8	0.0	0.0	4.8
	Angiosarcoma	73	51	47.1	41.2	9.8	2.0	52.9
	Dermatofibrosarcoma protuberans	21	16	25.0	68.8	6.3	0.0	75.0
	Ganglioneuroma	14	10	90.0	0.0	0.0	10.0	10.0
	Granular cell tumor	23	21	38.1	57.1	4.8	0.0	61.9
	Kaposi sarcoma	8	6	66.7	33.3	0.0	0.0	33.3
	Leiomyoma	50	40	57.5	42.5	0.0	0.0	42.5
	Leiomyosarcoma	87	77	26.0	31.2	22.1	20.8	74.0
	Liposarcoma	132	99	11.1	22.2	10.1	56.6	88.9
	Malignant peripheral nerve sheat tumor (MPNST)	13	12	83.3	8.3	8.3	0.0	16.7
	Myofibrosarcoma	26	25	52.0	8.0	4.0	36.0	48.0
	Neurofibroma	117	77	64.9	24.7	7.8	2.6	35.1
	Sarcoma, not otherwise specified (NOS)	75	68	38.2	25.0	8.8	27.9	61.8
	Paraganglioma	41	36	77.8	22.2	0.0	0.0	22.2
	Primitive neuroectodermal tumor (PNET)	23	20	35.0	50.0	10.0	5.0	65.0
	Rhabdomyosarcoma	7	7	28.6	0.0	0.0	71.4	71.4
	Schwannoma	121	97	12.4	47.4	23.7	16.5	87.6
	Synovial sarcoma	12	11	45.5	36.4	18.2	0.0	54.5
	Osteosarcoma	39	16	87.5	12.5	0.0	0.0	12.5
	Chondrosarcoma	43	29	41.4	6.9	10.3	41.4	58.6

### p16 immunostaining, tumor phenotype and prognosis

**Fig 4:**
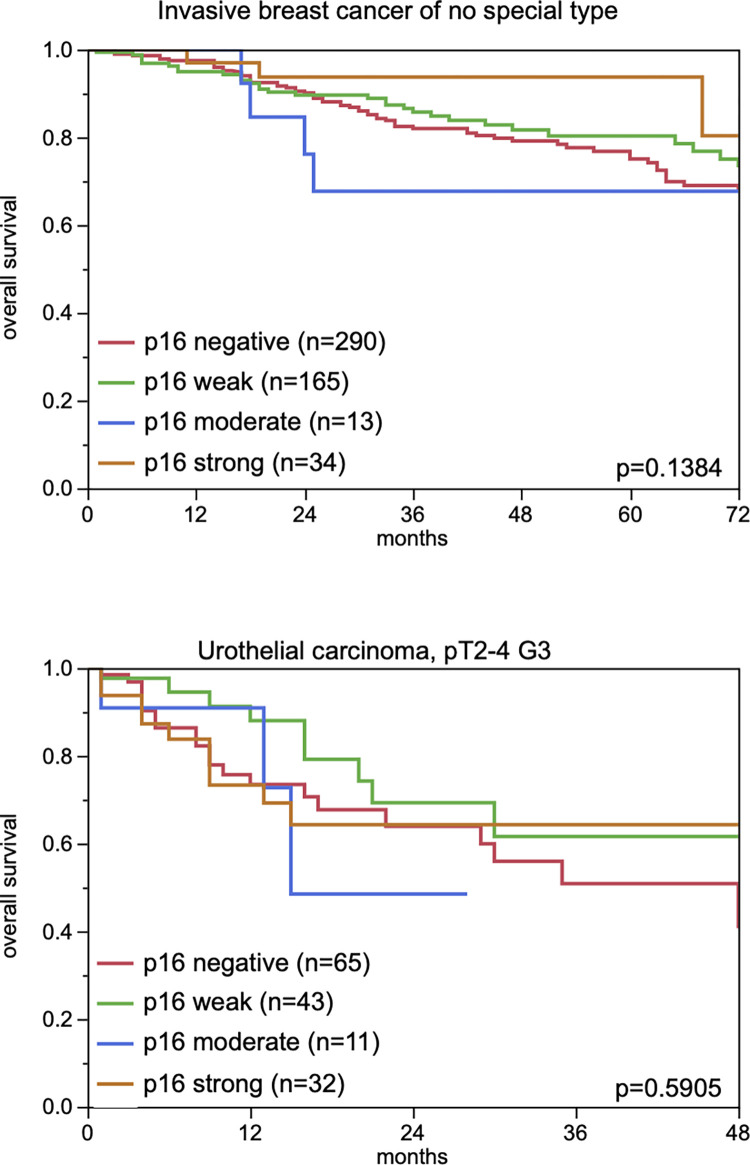
p16 immunostaining and overall survival in patients with invasive breast cancer of no special type and urothelial carcinoma (pT2-4; G3). *The numbers do not add to the total number of tumors with clinical follow-up data, since only cases with evaluable p16 staining are included.

A comparison of p16 expression with pT, pN, histologic grade, and patient prognosis in 128 analyzable vulvar carcinomas, 149 endometrioid endometrial carcinomas, 295 serous high grade ovarian carcinomas, 910 invasive breast carcinomas of no special type, 1245 urinary bladder carcinomas, 620 clear cell renal cell carcinomas, 414 germ cell tumors of the testis, 284 gastric adenocarcinomas, 396 pancreatic adenocarcinomas and 1365 colorectal adenocarcinomas revealed only few statistically significant associations (Table 2). Positive p16 immunostaining was associated with high pT category in urinary bladder carcinoma (p<0.0001) and gastric adenocarcinoma (p = 0.0212), and with high grade in invasive breast carcinoma of no special type (p<.0001). In colorectal adenocarcinoma, a significant association was found between p16 positivity (at least weak immunostaining) and MMR (mismatch repair) status, with a higher percentage of tumors showing positive p16 staining in the MMR-proficient group (56%) than in the MMR-deficient group (27%, p<0.0001). In cohorts of 502 invasive breast cancers and 151 urothelial carcinomas with clinical follow-up data, p16 immunostaining was unrelated to overall survival (Fig 4).

**Table 2:** p16 immunostaining and tumor phenotype.

		analyzable n*	p16 immunostaining				p value
			negative (%)	weak (%)	moderate (%)	strong (%)	
Vulvar carcinoma	all cancers	128	57.0	6.3	12.5	24.2	
	pT1	43	51.2	2.3	18.6	27.9	0.0880
	pT2	67	58.2	7.5	11.9	22.4	
	pT3-4	14	78.6	14.3	0.0	7.1	
	G1	16	68.8	12.5	18.8	0.0	0.1567
	G2	62	54.8	6.5	11.3	27.4	
	G3	30	56.7	6.7	13.3	23.3	
	pN0	68	50.0	4.4	16.2	29.4	0.1448
	pN+	37	64.9	10.8	8.1	16.2	
endometrioid endometrial carcinoma	all cancers	149	22.1	66.4	10.1	1.3	
	pT1	94	21.3	67.0	9.6	2.1	0.9198
	pT2	23	21.7	65.2	13.0	0.0	
	pT3-4	29	20.7	69.0	10.3	0.0	
	pN0	7	15.6	75.6	6.7	2.2	0.4994
	pN+	5	23.8	57.1	14.3	4.8	
serous high grade ovarian carcinoma	all cancers	295	15.2	12.9	49.2	22.7	
	pT1	20	5.0	0.0	70.0	25.0	0.1266
	pT2	38	18.4	13.2	42.1	26.3	
	pT3	208	15.9	14.4	47.1	22.6	
	pN0	65	15.4	9.2	53.9	21.5	0.1883
	pN1	138	10.9	18.8	44.2	26.1	
Invasive breast carcinoma of no special type	all cancers	910	61.1	30.2	3.0	5.7	
	pT1	452	61.5	32.7	1.8	4.0	0.0738
	pT2	358	62.8	25.7	3.6	7.8	
	pT3-4	70	61.4	28.6	2.9	7.1	
	G1	127.0	76.4	22.8	0.8	0.0	<0.0001
	G2	433.0	63.5	33.7	1.6	1.2	
	G3	349.0	52.7	28.4	5.4	13.5	
	pN0	468.0	65.5	27.4	3.1	4.1	0.8944
	pN+	343.0	60.7	30.6	2.2	6.6	
Urinary bladder carcinoma	all cancers	1245	45.7	32.5	8.4	13.4	
	pTa G2 low	145	19.3	80.0	0.7	0.0	<0.0001
	pTa G2 high	121	51.2	43.0	2.5	3.3	
	pTaG3	138	26.8	48.6	13.8	10.9	
	pT≥2 G3	780	51.3	20.3	10.0	18.5	
	normal urothelium	24	91.7	8.3	0.0	0.0	0.0465
	dysplasia	12	53.3	33.3	8.3	0.0	
Clear cell renal cell carcinoma	all cancers	620	98.9	1.0	0.1	0.0	
	pT1	365	98.9	1.1	0.0	0.0	0.4494
	pT2	63	100.0	0.0	0.0	0.0	
	pT3-4	187	98.4	1.1	0.5	0.0	
	ISUP 1	192	99.5	0.5	0.0	0.0	0.7094
	ISUP 2	204	99.0	1.0	0.0	0.0	
	ISUP 3	177	98.3	1.1	0.6	0.0	
	ISUP 4	38	97.4	2.6	0.0	0.0	
	pN0	98	99.0	1.0	0.0	0.0	0.5927
	pN≥1	15	100.0	0.0	0.0	0.0	
Germ cell tumors of the testis	all cancers	414	91.0	8.7	0.0	0.2	
	pT1	266	92.9	6.8	0.0	0.4	0.5059
	pT2	101	89.1	10.9	0.0	0.0	
	pT3	40	87.5	12.5	0.0	0.0	
Gastric carcinoma	all cancers	284	64.8	23.9	5.6	5.6	
	pT1-2	45	62.2	33.3	2.2	2.2	0.0212
	pT3	92	75.0	14.1	3.3	7.6	
	pT4	94	55.3	28.7	8.5	7.5	
	pN0	54	51.9	29.6	7.4	11.1	0.1350
	pN+	178	68.5	21.9	4.5	5.1	
Adenocarcinoma of the pancreas	all cancers	396	79.8	14.6	2.8	2.8	
	pT1	14	85.7	0.0	0.0	14.3	0.0486
	pT2	65	70.8	20.0	3.1	6.2	
	pT3	290	81.4	13.8	3.1	1.7	
	pT4	26	80.8	19.2	0.0	0.0	
	G1	13	69.2	23.1	0.0	7.7	0.8606
	G2	271	79.3	14.8	3.3	2.6	
	G3	87	80.5	14.9	2.3	2.3	
	pN0	84	82.1	10.7	3.6	3.6	0.6210
	pN+	310	79.0	15.8	2.6	2.6	
	R0	216	74.5	16.7	4.6	4.2	0.0007
	R1	155	87.1	11.6	0.0	1.3	
Colorectal adenocarcinoma	all cancers	1365	48.2	48.6	2.6	0.6	
	pT1	57	47.4	52.6	0.0	0.0	0.4898
	pT2	271	45.8	50.9	3.0	0.4	
	pT3	745	48.2	48.2	3.1	0.5	
	pT4	280	50.0	47.5	1.4	1.1	
	pN0	698	48.3	48.6	2.6	0.6	0.9965
	pN+	639	47.7	49.0	2.7	0.6	
	MMR proficient	71	73.2	25.4	0.0	1.4	<0.0001
	MMR deficient	983	44.2	52.6	2.7	0.5	

Abbreviation: pT: pathological tumor stage, pN: pathological lymph node status, G: grade, ISUP: International Society of Urological Pathology, R: resection margin, MMR: mismatch repair.

*Numbers do not always add up to the total number in the different categories because of cases with missing data.

### p16 immunostaining and HPV-status

HPV analysis of 535 squamous cell carcinomas of different sites of origin revealed 244 HPV positive cases (45.6%). Among these, 98.0% were high risk type (HPV type 16, 18, 33, 35, 45, 58), 1.6% intermediate risk type (HPV type 56, 67, 73) and 0.4% low risk type (HPV type 6). The comparison of HPV status and p16 staining revealed a strong but not perfect association between these parameters (Table 3). HPV was detected in 80.4% of 163 tumors with strong, 62.3% of 42 tumors with moderate, 25.9% of 42 tumors with weak p16 positivity, but also in 20.6% of 250 p16 negative cancers (p<0.0001). The association between p16 expression and HPV status varied between the organs of tumor origin and was particularly strong in squamous cell carcinomas of the cervix, squamous cell carcinoma of the penis and squamous cell carcinoma of the pharynx (p<0.0001 each). The statistical association between p16 expression and HPV status was particularly weak in squamous cell carcinomas of the larynx and of the vulva. It is of note, however, that both HPV negative cases with strong p16 positivity, and HPV positive cases with negative p16 staining were found in almost all tumor entities. Only cervical and skin cancer lacked HPV positive but p16 negative cases.

**Table 3:** Association between p16 immunostaining and HPV status.

	p16 status	n	HPV status		
			negative	positive	
All cancers	negative	253	79.4	20.6	<0.0001
	weak	54	74.1	25.9	
	moderate	53	37.7	62.3	
	strong	163	19.6	80.4	
Oral squamous cell carcinoma	negative	55	89.1	10.9	0.0017
	weak	3	100.0	0.0	
	moderate	4	100.0	0.0	
	strong	9	33.3	66.7	
Squamous cell carcinoma of the pharynx	negative	25	72.0	28.0	<0.0001
	weak	4	25.0	75.0	
	moderate	4	25.0	75.0	
	strong	17	5.9	94.1	
Squamous cell carcinoma of the larynx	negative	47	83.0	17.0	0.3388
	weak	2	100.0	0.0	
	moderate	2	50.0	50.0	
	strong	4	100.0	0.0	
Squamous cell carcinoma of the cervix	negative	4	100.0	0.0	<0.0001
	weak	2	0.0	100.0	
	moderate	12	0.0	100.0	
	strong	57	7.0	93.0	
Squamous cell carcinoma of the vagina	negative	16	68.8	31.3	0.0077
	weak	2	100.0	0.0	
	moderate	4	0.0	100.0	
	strong	7	28.6	71.4	
Squamous cell carcinoma of the vulva	negative	44	72.7	27.3	0.0571
	weak	12	83.3	16.7	
	moderate	6	33.3	66.7	
	strong	13	46.2	53.8	
Squamous cell carcinoma of penis	negative	34	67.6	32.4	<0.0001
	weak	6	16.7	83.3	
	moderate	3	0.0	100.0	
	strong	28	14.3	85.7	
Squamous cell carcinoma of the skin	negative	19	100.0	0.0	0.0951
	weak	9	100.0	0.0	
	moderate	2	50.0	50.0	
	strong	6	100.0	0.0	
Squamous cell carcinoma of the anal canal	negative	6	50.0	50.0	0.0723
	weak	2	0.0	100.0	
	moderate	5	0.0	100.0	
	strong	22	9.1	90.9	

## Discussion

The successful analysis of 11,759 cancers and 76 normal tissue types revealed that–as compared to normal tissues—p16 is often upregulated in cancers. While the normal tissue analysis demonstrated a moderate to strong p16 immunostaining in only few tissues, a strong p16 positivity was found in many tumors. Although p16 is a known tumor suppressor gene, upregulation can occur directly as a consequence of an altered state of the interaction partner pRb [31] or indirectly through pathway crosstalk with p53 (reviewed in [32]). Considering that p53 is the most frequently mutated tumor suppressor gene in cancer [33], that p53 inactivation can also occur in the absence of p53 gene mutations (reviewed in [34]), and that alterations of Rb (reviewed in [35]) and other p16 interaction partners such as CDK4 (cyclin dependent kinase 4) [36] are also common in cancer, the high rate of p16 upregulation is not a surprise. Our data also revealed a correlation between p16 staining and microsatellite instability in colorectal adenocarcinoma. In the microsatellite-stable group a higher percentage of tumors showed positive p16 staining than in the microsatellite-instable group. This may be explained by the known inverse relationship of p53 alterations (a well-established cause for p16 upreagulation) and MSI in colorectal cancer. Moreover, it has been shown that microsatellite instability leads to increased methylation of the p16 gene which may lead to reduced p16 expression or at least hinder p16 upregulation [37]. The results of our study do not exclude that p16 expression can also be reduced in some cancers as described in previous studies [15,38–41]. However, an entirely different experimental approach than the one selected for this study, with a much higher antibody concentration and a more sensitive staining protocol would be required to demonstrate reduced expression.

That a minimum of one case with at least a moderate p16 positivity was found in 100 of our 124 (80.6%) analyzed cancer types demonstrates that p16 immunostaining offers only limited support for defining a tumor’s site of origin. According to our data, there are only few occasions, where p16 immunostaining can assist in diagnosing the right tumor type. This applies for example—as previously suggested [42]—in the differentiation of high grade endometrial from serous carcinoma of the endometrium. In our study 40.6% of serous endometrial carcinoma showed strong p16 immunostaining in comparison to 5.1% strong p16 positivity in endometroid endometrial carcinoma.

In normal tissues, moderate to high p16 immunostaining was only consistently seen in islets of Langerhans of the pancreas and in the pituitary gland. p16 immunostaining was largely absent in tissues which are prone to develop cancer such as urothelium and squamous cell epithelium of various sites. Given the high rate of p16 overexpression in various cancers developing from p16 negative cells, p16 immunostaining may serve as a parameter that might indicate malignancy in some organs. Based on our findings that might for example apply for liposarcoma (56.6% strong positive, vs negative in normal fat), leiomyosarcoma (21% strong positive, vs 0% strong positive cases in leiomyoma) or urothelial dysplasia (46.7% positive, vs negative in normal urothelium). The high utility of immunohistochemical p16 analysis in assessing cervical biopsies is based on the fact that almost all neoplasias in this location are due to HPV infection [43]. The imperfect correlation of p16 expression and HPV infection found in our analysis of 497 squamous cell carcinomas of different origins suggest low reliability for using p16 immunostaining as a surrogate for HPV infection in other cancer types, however. Of note, in our study HPV could be detected in 20.6% of cases with absent p16 immunostaining. On the other hand, it is not surprising, that moderate to strong p16 positivity was found in 25–100% (average 30.7%) of HPV negative extra-genital squamous cell carcinomas, given the interaction of p16 with several important pathways. That aberrant p16 expression was not only seen in endothelial cells of a few cancers, but also in rare instances in endothelial cells in non-neoplastic tissue and in fibroblasts of the tumor stroma demonstrates, that substantial p16 upregulation can occasionally also occur in non-neoplastic tissue proliferation.

Our highly standardized analysis of 11,759 tumors from 124 different tumor entities enabled us to clarify the relative importance of p16 expression across tumor entities and to generate a ranking list according to the p16 positivity rate (Fig 3). It is of note, that many of the top ranked p16 positive tumor entities such as small cell neuroendocrine carcinoma of the bladder, Merkel cell carcinoma, small cell carcinoma of the lung and small cell neuroendocrine carcinoma of the prostate exhibit neuroendocrine differentiation. This observation is in line with data from an earlier study showing, that loss of Rb function, which can cause overexpression of p16, leads to neuroendocrine hypercellularity in the lung [44]. Although we are unaware of a specific role of p16 in neuroendocrine cellular functions, it is conspicuous that our normal tissue analysis had also identified the highest expression in neuroendocrine/endocrine cells of islets of Langerhans in the pancreas, and the adenohypophysis. Moreover, it is possible that the scattered p16 positive cells in the gastrointestinal tract also represent endocrine cells. Several other studies have also shown p16 overexpression in various neuroendocrine neoplasms of different origin [45–47].

**Fig 5:**
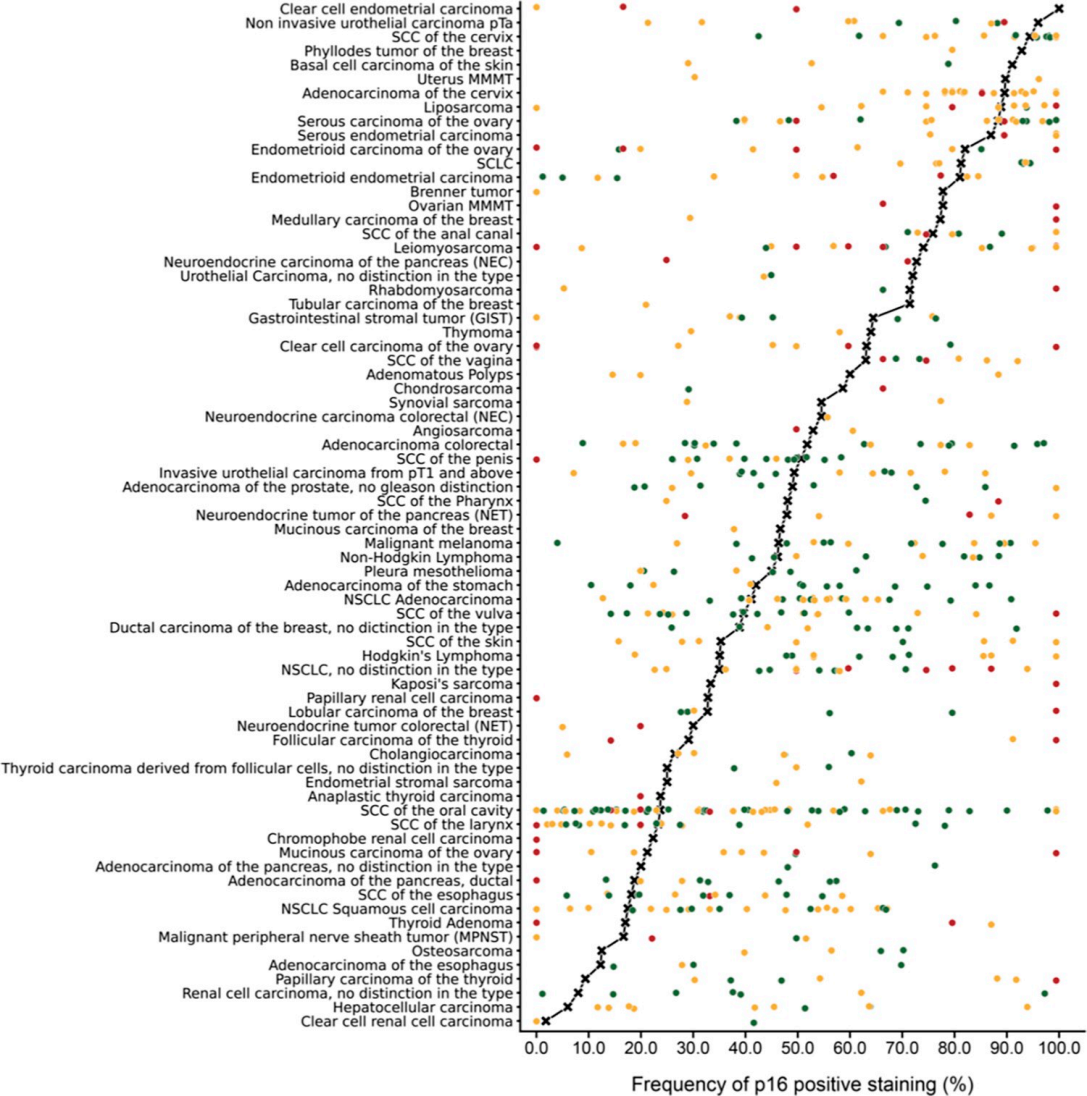
Graphical representation of p16 data from this study (marked with a cross) in comparison with data from existing literature (marked with dots). In order to simplify the figure the percentage of weak, moderate and strong staining was merged. Red dots are used for previous studies involving 1–9 cases, yellow dots for studies involving 10–50 cases and green dots for studies involving >50 cases. All studies are quoted in a list of references in S1 Table.

More than 3000 studies have previously analyzed p16 expression in tumors by immunohistochemistry. The summary of the results of 448 of these studies in Fig 5 demonstrates, that highly discrepant data on the prevalence of p16 positivity exist for many tumor entities. This wide range of published p16 positivity rates makes it difficult to assess the potential significance of p16 immunohistochemistry in individual tumor entities and may also be responsible for conflicting data on the potential prognostic and diagnostic relevance of p16 expression in such tumor entities. That our own analyses of associations between clinico-pathological parameters of cancer aggressiveness and p16 expression mostly revealed only weak or even no associations seems to suggest, that p16 overexpression is not a feature that is dramatically linked to lethal cancer cell properties.

In summary, these results provide a comprehensive overview on p16 expression in human normal tissues and cancers. The absence of a significant p16 expression in most normal tissues in combination with a high frequency of p16 overexpression in cancers of all types demonstrates a significant role of p16 in cancer biology and suggest a general utility of p16 immunohistochemistry as a potential aid to diagnose malignancy. The lack of striking associations of p16 immunostaining with clinico-pathological parameters for cancer aggressiveness in most analyzed cancer types argues against a major prognostic impact of p16 protein expression, however.

## Supporting information

S1 Table List of studies used to generate Fig 5. (XLSX)

S2 Table p16 positive and p16 negative normal tissues and associated tumor types. (XLSX)
