# Correction: High prevalence of p16 staining in malignant tumors

**DOI:** 10.1371/journal.pone.0318271

**Published:** 2025-01-24

**Authors:** Noémi De Wispelaere, Sebastian Dwertmann Rico, Marcus Bauer, Andreas M. Luebke, Martina Kluth, Franziska Büscheck, Claudia Hube-Magg, Doris Höflmayer, Natalia Gorbokon, Sören Weidemann, Katharina Möller, Christoph Fraune, Christian Bernreuther, Ronald Simon, Christian Kähler, Anne Menz, Andrea Hinsch, Frank Jacobsen, Patrick Lebok, Till Clauditz, Guido Sauter, Ria Uhlig, Waldemar Wilczak, Stefan Steurer, Eike Burandt, Rainer Krech, David Dum, Till Krech, Andreas Marx, Sarah Minner

In Table 1, there are errors in the values indicated to the columns of p16 immunostaining of rows “Non-invasive papillary urothelial carcinoma, pTa G2 low grade”, “Non-invasive papillary urothelial carcinoma, pTa G2 high grade” and “Non-invasive papillary urothelial carcinoma, pTa G3” under the category of Tumors of the urinary system. Also, the “(neg. = negative, mod. = moderate, pos. = positive).” is missing from its caption. Please see the correct Table 1 and its complete caption here.

**Table 1:** p16 immunostaining in human tumors (neg. = negative, mod. = moderate, pos. = positive).

	tumor entity	on TMA (n)	p16 immunostaining					
			analy-zable (n)	neg. (%)	weak (%)	mod. (%)	strong (%)	pos. (%)
**Tumors of the skin**	Pilomatrixoma	35	28	32.1	64.3	3.6	0.0	67.9
	Basal cell carcinoma	88	67	9.0	68.7	20.9	1.5	91.0
	Benign nevus	29	25	4.0	60.0	20.0	16.0	96.0
	Squamous cell carcinoma of the skin	90	85	64.7	20.0	5.9	9.4	35.3
	Malignant melanoma	48	41	53.7	19.5	19.5	7.3	46.3
	Merkel cell carcinoma	46	44	2.3	2.3	9.1	86.4	97.7
**Tumors of the head and neck**	Squamous cell carcinoma of the larynx	110	90	76.7	13.3	4.4	5.6	23.3
	Squamous cell carcinoma of the pharynx	60	52	51.9	7.7	7.7	32.7	48.1
	Oral squamous cell carcinoma (floor of the mouth)	130	114	76.3	7.9	3.5	12.3	23.7
	Pleomorphic adenoma of the parotid gland	50	33	6.1	81.8	12.1	0.0	93.9
	Warthin tumor of the parotid gland	49	41	2.4	87.8	9.8	0.0	97.6
	Basal cell adenoma of the salivary gland	15	13	0.0	100.0	0.0	0.0	100.0
**Tumors of the lung, pleura** **and thymus**	Squamous cell carcinoma of the lung	77	40	82.5	5.0	10.0	2.5	17.5
	Adenocarcinoma of the lung	200	107	58.9	27.1	10.3	3.7	41.1
	Small cell carcinoma of the lung	20	16	18.8	12.5	6.3	62.5	81.3
	Mesothelioma, epitheloid	39	30	76.7	23.3	0.0	0.0	23.3
	Mesothelioma, other types	76	63	63.5	12.7	0.0	42.9	55.6
	Thymoma	29	25	36.0	60.0	4.0	0.0	64.0
**Tumors of the female genital** **tract**	Squamous cell carcinoma of the vagina	78	73	37.0	11.0	9.6	42.5	63.0
	Squamous cell carcinoma of the vulva	130	116	60.3	12.1	8.6	19.0	39.7
	Squamous cell carcinoma of the cervix	130	125	5.6	4.0	11.2	79.2	94.4
	Adenocarcinoma of the cervix uteri	50	48	10.4	52.1	22.9	14.6	89.6
	Endometrioid endometrial carcinoma	236	195	19.0	63.6	12.3	5.1	81.0
	Endometrial serous carcinoma	82	69	13.0	26.1	20.3	40.6	87.0
	Carcinosarcoma of the uterus	48	39	10.3	20.5	38.5	30.8	89.7
	Endometrial carcinoma, high grade, G3	13	8	12.5	62.5	12.5	12.5	87.5
	Endometrial clear cell carcinoma	8	5	0.0	80.0	20.0	0.0	100.0
	Endometrial stromal sarcoma	12	12	75.0	16.7	0.0	8.3	25.0
	Endometrioid carcinoma of the ovary	115	89	18.0	47.2	19.1	15.7	82.0
	Serous carcinoma of the ovary	567	446	11.7	24.0	16.4	48.0	88.3
	Mucinous carcinoma of the ovary	97	66	78.8	18.2	3.0	0.0	21.2
	Clear cell carcinoma of the ovary	54	38	36.8	47.4	15.8	0.0	63.2
	Carcinosarcoma of the ovary	47	36	22.2	30.6	19.4	27.8	77.8
	Brenner tumor	9	9	22.2	66.7	11.1	0.0	77.8
**Tumors of the breast**	Invasive breast carcinoma of no special type	1387	960	61.1	29.5	3.6	5.7	38.9
	Lobular carcinoma of the breast	294	168	67.3	30.4	1.2	1.2	32.7
	Medullary carcinoma of the breast	26	22	22.7	13.6	13.6	50.0	77.3
	Tubular carcinoma of the breast	27	14	28.6	71.4	0.0	0.0	71.4
	Mucinous carcinoma of the breast	58	30	53.3	40.0	6.7	0.0	46.7
	Phyllodes tumor of the breast	50	42	7.1	71.4	14.3	7.1	92.9
**Tumors of the digestive system**	Adenomatous polyp, low-grade dysplasia	50	49	53.1	44.9	2.0	0.0	46.9
	Adenomatous polyp, high-grade dysplasie	50	49	26.5	63.3	10.2	0.0	73.5
	Adenocarcinoma of the colon	1882	1434	48.2	48.6	2.6	0.6	51.8
	Adenocarcinoma of the small intestine	10	10	80.0	0.0	10.0	10.0	20.0
	Gastric adenocarcinoma, diffuse type	176	101	54.5	28.7	10.9	5.9	45.5
	Gastric adenocarcinoma, intestinal type	174	127	59.1	23.6	7.1	10.2	40.9
	Gastric adenocarcinoma, mixed type	62	45	62.2	28.9	0.0	8.9	37.8
	Adenocarcinoma of the esophagus	133	57	87.7	1.8	1.8	8.8	12.3
	Squamous cell carcinoma of the esophagus	124	44	81.8	0.0	4.5	13.6	18.2
	Squamous cell carcinoma of the anal canal	91	87	24.1	4.6	10.3	60.9	75.9
	Cholangiocarcinoma	130	102	73.5	20.6	3.9	2.0	26.5
	Hepatocellular carcinoma	50	50	94.0	6.0	0.0	0.0	6.0
	Ductal adenocarcinoma of the pancreas	612	410	81.2	13.2	3.2	2.4	18.8
	Pancreatic/Ampullary adenocarcinoma	89	52	69.2	21.2	5.8	3.8	30.8
	Acinar cell carcinoma of the pancreas	13	12	75.0	8.3	8.3	8.3	25.0
	Gastrointestinal stromal tumor (GIST)	50	45	35.6	40.0	8.9	15.6	64.4
**Tumors of the urinary system**	Non-invasive papillary urothelial carcinoma, pTa G2 low grade	177	116	19.3	80.0	0.7	0.0	80.7
	Non-invasive papillary urothelial carcinoma, pTa G2 high grade	141	106	51.2	43.0	2.5	3.3	48.8
	Non-invasive papillary urothelial carcinoma, pTa G3	187	132	26.8	48.6	13.8	10.9	73.2
	Urothelial carcinoma, pT2-4 G3	1214	732	50.7	20.4	10.1	18.9	49.3
	Small cell neuroendocrine carcinoma of the bladder	18	18	0.0	0.0	0.0	100.0	100.0
	Sarcomatoid urothelial carcinoma	25	24	54.2	12.5	0.0	33.3	45.8
	Clear cell renal cell carcinoma	858	648	98.2	1.7	0.2	0.0	1.8
	Papillary renal cell carcinoma	255	129	67.2	31.3	1.6	0.0	32.8
	Clear cell (tubulo) papillary renal cell carcinoma	21	13	92.9	7.1	0.0	0.0	7.1
	Chromophobe renal cell carcinoma	131	80	77.7	22.3	0.0	0.0	22.3
	Oncocytoma	177	128	95.5	4.5	0.0	0.0	4.5
**Tumors of the male genital** **organs**	Adenocarcinoma of the prostate, Gleason 3+3	83	63	92.1	6.3	1.6	0.0	7.9
	Adenocarcinoma of the prostate, Gleason 4+4	80	64	67.2	31.3	1.6	0.0	32.8
	Adenocarcinoma of the prostate, Gleason 5+5	85	61	63.9	36.1	0.0	0.0	36.1
	Adenocarcinoma of the prostate (recurrence)	330	284	35.6	57.0	2.5	4.9	64.4
	Small cell neuroendocrine carcinoma of the prostate	17	16	6.3	18.8	12.5	62.5	93.8
	Seminoma	620	454	90.3	9.3	0.2	0.2	9.7
	Embryonal carcinoma of the testis	50	41	82.9	14.6	2.4	0.0	17.1
	Yolk sac tumor	50	36	80.6	19.4	0.0	0.0	19.4
	Teratoma	50	36	88.9	5.6	2.8	2.8	11.1
	Squamous cell carcinoma of the penis	80	75	49.3	8.0	4.0	38.7	50.7
**Tumors of endocrine organs**	Adenoma of the thyroid gland	50	47	83.0	17.0	0.0	0.0	17.0
	Papillary thyroid carcinoma	114	96	90.6	9.4	0.0	0.0	9.4
	Follicular thyroid carcinoma	392	333	70.9	26.4	2.4	0.3	29.1
	Medullary thyroid carcinoma	158	130	87.7	11.5	0.8	0.0	12.3
	Anaplastic thyroid carcinoma	107	80	76.3	22.5	1.3	0.0	23.8
	Adrenal cortical adenoma	45	42	66.7	2.4	7.1	23.8	33.3
	Adrenal cortical carcinoma	26	26	26.9	23.1	19.2	30.8	73.1
	Phaeochromocytoma	50	50	58.0	38.0	4.0	0.0	42.0
	Appendix, neuroendocrine tumor (NET)	22	13	38.5	53.8	7.7	0.0	61.5
	Colorectal, neuroendocrine tumor (NET)	10	10	70.0	30.0	0.0	0.0	30.0
	Ileum, neuroendocrine tumor (NET)	49	47	76.6	23.4	0.0	0.0	23.4
	Lung, neuroendocrine tumor (NET)	19	17	82.4	17.6	0.0	0.0	17.6
	Pancreas, neuroendocrine tumor (NET)	102	96	52.1	40.6	5.2	2.1	47.9
	Colorectal, neuroendocrine carcinoma (NEC)	11	11	45.5	0.0	27.3	27.3	54.5
	Gallbladder, neuroendocrine carcinoma (NEC)	4	4	25.0	0.0	75.0	0.0	75.0
	Pancreas, neuroendocrine carcinoma (NEC)	13	11	27.3	36.4	18.2	18.2	72.7
**Tumors of haemotopoetic** **and lymphoid organs**	Hodgkin Lymphoma	103	77	64.9	23.4	10.4	1.3	35.1
	Non-Hodgkin Lymphoma	62	52	53.8	44.2	1.9	0.0	46.2
	Small lymphocytic lymphoma, B-cell type (B-SLL/B-CLL)	50	48	54.2	45.8	0.0	0.0	45.8
	Diffuse large B cell lymphoma (DLBCL)	114	110	63.6	29.1	5.5	1.8	36.4
	Follicular lymphoma	88	84	58.3	41.7	0.0	0.0	41.7
	T-cell Non Hodgkin lymphoma	24	24	58.3	33.3	8.3	0.0	41.7
	Mantle cell lymphoma	18	18	83.3	16.7	0.0	0.0	16.7
	Marginal zone lymphoma	16	15	80.0	20.0	0.0	0.0	20.0
	Diffuse large B-cell lymphoma (DLBCL) in the testis	16	15	80.0	13.3	6.7	0.0	20.0
	Burkitt lymphoma	5	4	75.0	25.0	0.0	0.0	25.0
**Tumors of soft tissue and bone**	Tenosynovial giant cell tumor	45	43	9.3	88.4	2.3	0.0	90.7
	Angiomyolipoma	91	84	95.2	4.8	0.0	0.0	4.8
	Angiosarcoma	73	51	47.1	41.2	9.8	2.0	52.9
	Dermatofibrosarcoma protuberans	21	16	25.0	68.8	6.3	0.0	75.0
	Ganglioneuroma	14	10	90.0	0.0	0.0	10.0	10.0
	Granular cell tumor	23	21	38.1	57.1	4.8	0.0	61.9
	Kaposi sarcoma	8	6	66.7	33.3	0.0	0.0	33.3
	Leiomyoma	50	40	57.5	42.5	0.0	0.0	42.5
	Leiomyosarcoma	87	77	26.0	31.2	22.1	20.8	74.0
	Liposarcoma	132	99	11.1	22.2	10.1	56.6	88.9
	Malignant peripheral nerve sheat tumor (MPNST)	13	12	83.3	8.3	8.3	0.0	16.7
	Myofibrosarcoma	26	25	52.0	8.0	4.0	36.0	48.0
	Neurofibroma	117	77	64.9	24.7	7.8	2.6	35.1
	Sarcoma, not otherwise specified (NOS)	75	68	38.2	25.0	8.8	27.9	61.8
	Paraganglioma	41	36	77.8	22.2	0.0	0.0	22.2
	Ewing sarcoma	23	20	35.0	50.0	10.0	5.0	65.0
	Rhabdomyosarcoma	7	7	28.6	0.0	0.0	71.4	71.4
	Schwannoma	121	97	12.4	47.4	23.7	16.5	87.6
	Synovial sarcoma	12	11	45.5	36.4	18.2	0.0	54.5
	Osteosarcoma	39	16	87.5	12.5	0.0	0.0	12.5
	Chondrosarcoma	43	29	41.4	6.9	10.3	41.4	58.6

In the S2 Table, the values in the rows “Non-invasive papillary urothelial carcinoma, pTa G2 low grade”, “Non-invasive papillary urothelial carcinoma, pTa G2 high grade” and Non-invasive papillary urothelial carcinoma, pTa G3 of the organ urinary system are incorrect. The authors have provided the correct version of S2 Table here.

**S2 Table:** p16 positive and p16 negative normal tissues and associated tumor types.

Nomal tissues			Tumor tissues	
Organ	Normal cell type	Immunostaining intensity	Tumor type	% p16 positiv
Pancreas	Islets of Langerhans	Strong	Acinar cell carcinoma of the pancreas	25.0
			Ductal adenocarcinoma of the pancreas	18.8
			Pancreatic/Ampullary adenocarcinoma	30.8
			Pancreas, neuroendocrine tumor (NET)	47.9
			Pancreas, neuroendocrine carcinoma (NEC)	72.7
Brain	Adenohypophysis	Strong	Schwannoma	87.6
Stomach	Gastrointestinal mucosa	Weak to moderate	Gastrointestinal stromal tumor (GIST)	64.4
			Gastric adenocarcinoma, diffuse type	45.5
			Gastric adenocarcinoma, intestinal type	40.9
			Gastric adenocarcinoma, mixed type	37.8
Salivary gland	Epithelium of ducts	Weak to moderate	Basal cell adenoma of the salivary gland	100.0
Prostate	Glandular cells	Weak to moderate	Adenocarcinoma of the prostate, Gleason 3+3	7.9
			Adenocarcinoma of the prostate, Gleason 4+4	32.8
			Adenocarcinoma of the prostate, Gleason 5+5	36.1
			Adenocarcinoma of the prostate (recurrence)	64.4
			Small cell neuroendocrine carcinoma of the prostate	93.8
Breast	Glandular cells	Weak to moderate	Invasive breast carcinoma of no special type	38.9
			Lobular carcinoma of the breast	32.7
			Medullary carcinoma of the breast	77.3
			Tubular carcinoma of the breast	71.4
			Mucinous carcinoma of the breast	46.7
			Phyllodes tumor of the breast	92.9
Adrenal gland	Glandular cells	Weak to moderate	Adrenal cortical adenoma	33.3
			Adrenal cortical carcinoma	73.1
Uterus	Endometrium	Weak to moderate	Endometrioid endometrial carcinoma	81.0
			Endometrial serous carcinoma	87.0
			Endometrial carcinoma, high grade, G3	87.5
			Endometrial clear cell carcinoma	100.0
			Endometrial stromal sarcoma	25.0
			Endometrioid carcinoma of the ovary	82.0
Lymphatic tissues		Weak to moderate	Hodgkin Lymphoma	35.1
			Non-Hodgkin Lymphoma	46.2
			Small lymphocytic lymphoma, B-cell type (B-SLL/B-CLL)	45.8
			Diffuse large B cell lymphoma (DLBCL)	36.4
			Follicular lymphoma	41.7
			T-cell Non Hodgkin lymphoma	41.7
			Mantle cell lymphoma	16.7
			Marginal zone lymphoma	20.0
			Diffuse large B-cell lymphoma (DLBCL) in the testis	20.0
			Burkitt lymphoma	25.0
Skin	Muscular wall, hair follicles and sebaceous glands	negative	Pilomatrixoma	67.9
	Transitional mucosa	negative	Basal cell carcinoma	91.0
			Benign nevus	96.0
			Squamous cell carcinoma of the skin	35.3
			Malignant melanoma	46.3
			Merkel cell carcinoma	97.7
Head and Neck	Muscular wall and mucosa	negative	Squamous cell carcinoma of the larynx	23.3
			Squamous cell carcinoma of the pharynx	48.1
			Oral squamous cell carcinoma (floor of the mouth)	23.7
	Squamous epithelium of the esophagus	negative	Adenocarcinoma of the esophagus	12.3
			Squamous cell carcinoma of the esophagus	18.3
Digestive system	Muscular wall of the colon	negative	Adenomatous polyp, low-grade dysplasia	46.9
			Adenomatous polyp, high-grade dysplasie	73.5
			Adenocarcinoma of the colon	51.8
	Brunner gland of the duodenum	negative	Adenocarcinoma of the small intestine	20.0
	Transitional mucosa of the analy canal	negative	Squamous cell carcinoma of the anal canal	75.9
	Muscular wall of the appendix	negative	Appendix, neuroendocrine tumor (NET)	61.5
	Epithelium of the gallbladder	negative	Gallbladder, neuroendocrine carcinoma (NEC)	75.0
	Epithelium of the liver	negative	Hepatocellular carcinoma	6.0
			Non-invasive papillary urothelial carcinoma, pTa G2 low grade	80.7
Urinary system	Urothelium of the urinary bladder	negative	Non-invasive papillary urothelial carcinoma, pTa G2 high grade	48.8
			Non-invasive papillary urothelial carcinoma, pTa G3	73.2
			Urothelial carcinoma, pT2-4 G3	49.3
			Small cell neuroendocrine carcinoma of the bladder	100.0
	Kidney pelvis	negative	Clear cell renal cell carcinoma	1.8
	Urothelium of the kidney pelvis	negative	Papillary renal cell carcinoma	32.8
	Cortex and medulla of the kidney	negative	Clear cell (tubulo) papillary renal cell carcinoma	7.1
			Chromophobe renal cell carcinoma	22.3
			Oncocytoma	4.5
Male genital organs	Corpus spongiosum of the penis	negative	Squamous cell carcinoma of the penis	50.7
	Testis	negative	Seminoma	9.7
	Epididymis	negative	Embryonal carcinoma of the testis	17.1
			Yolk sac tumor	19.4
			Teratoma	11.1
Female genital tract	Ovarian stroma	negative	Endometrioid carcinoma of the ovary	82.0
	Fallopian tube mucosa	negative	Serous carcinoma of the ovary	88.3
			Mucinous carcinoma of the ovary	21.2
			Clear cell carcinoma of the ovary	63.2
			Carcinosarcoma of the ovary	77.8
			Brenner tumor	77.8
	Epithelium of the endocervix	negative	Squamous cell carcinoma of the cervix	94.4
	Transitional mucosa of the ectocervix	negative	Adenocarcinoma of the cervix uteri	89.6
	Myometrium of the uterus	negative	Carcinosarcoma of the uterus	89.7
Soft tissues	Striated muscle	negative	Leiomyoma	42.5
			Leiomyosarcoma	74.0
			Rhabdomyosarcoma	71.4
	Fat	negative	Liposarcoma	88.9

In Figs 3 and 4, the ranking order of tumor types is incorrect. Please see the correct Figs 3 and 4 here.

**Fig 3:**
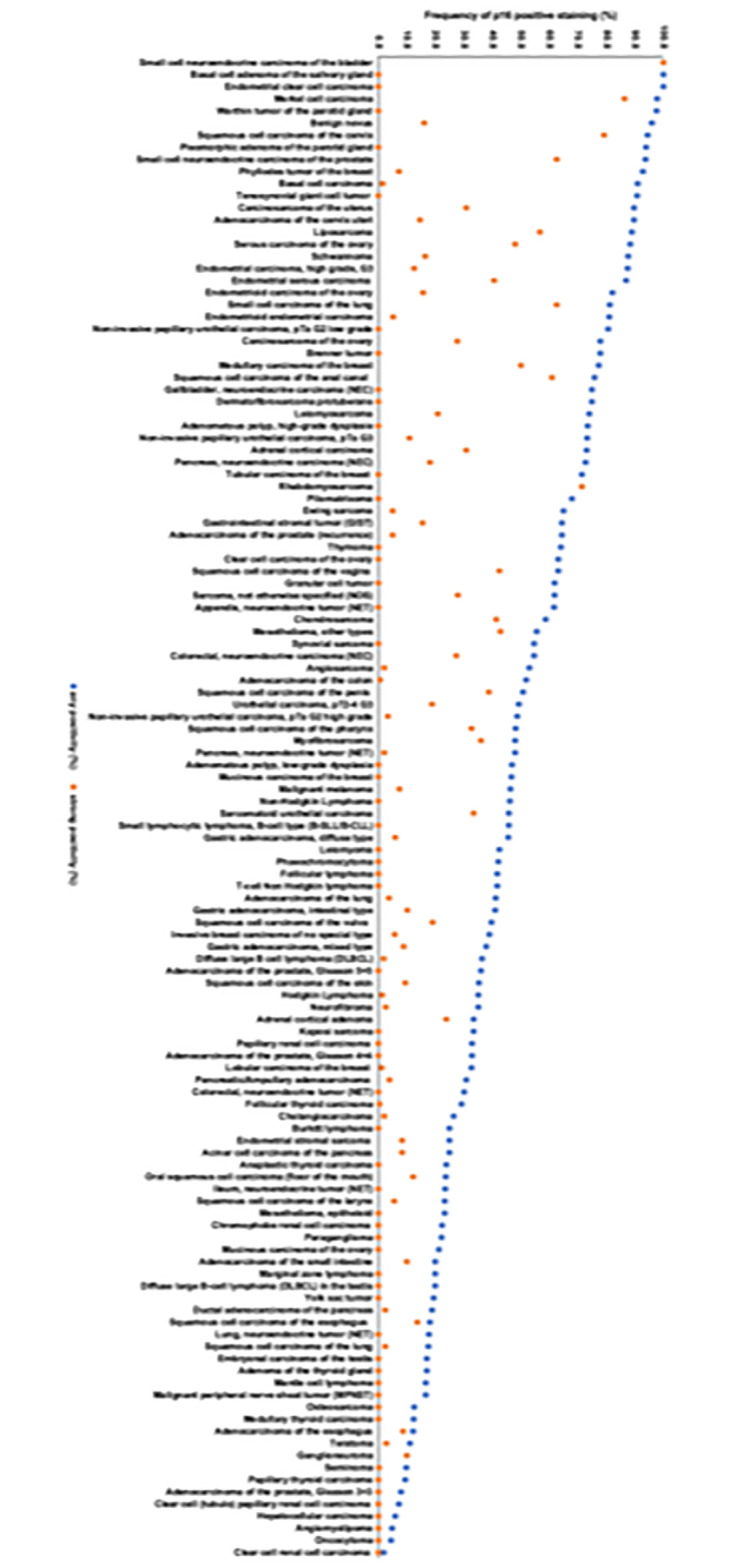
Ranking order of p16 immunostaining in human tumors. Both the frequency of positive cases (blue dots) and the frequency of strongly positive cases (orange dots) is shown.

**Fig 5:**
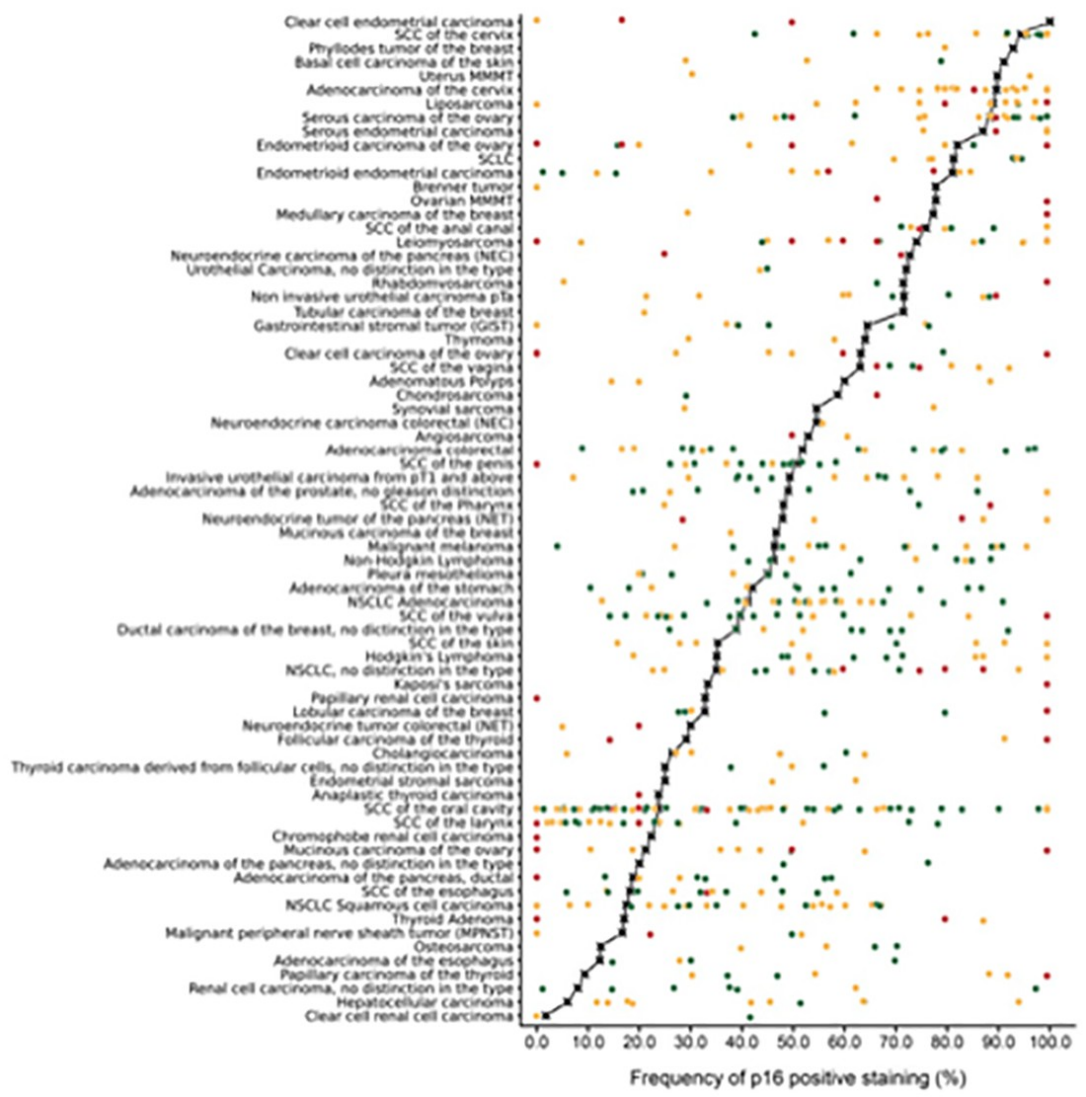
Graphical representation of p16 data from this study (marked with a cross) in comparison with data from existing literature (marked with dots). In order to simplify the figure the percentage of weak, moderate and strong staining was merged. Red dots are used for previous studies involving 1–9 cases, yellow dots for studies involving 10–50 cases and green dots for studies involving >50 cases. All studies are quoted in a list of references in S1 Table.

There is an error in fourth sentence of the Abstract section. The correct sentence is: In cancer, highest positivity rates were observed in uterine cervix squamous cell carcinomas (94.4%), Merkel cell carcinoma (97.7%), and small cell carcinomas of various sites of origin (54.5%-100%).
